# Behavioral barriers in the management of spinal muscular atrophy: The role of procrastination, regret, and burnout

**DOI:** 10.1371/journal.pone.0350643

**Published:** 2026-06-02

**Authors:** Jorge Maurino, Laura Carrera-García, Paz Castro-Fernández, Ignacio Málaga, Andrés Nascimento, Gustavo Saposnik

**Affiliations:** 1 Medical Department, Roche Farma, Madrid, Spain; 2 Neuromuscular Unit, Department of Neurology, Hospital Sant Joan de Déu, Barcelona, Spain; 3 Group of Applied Research in Neuromuscular Diseases, Institut de Recerca Sant Joan de Déu, Barcelona, Spain; 4 Department of Pediatric Neurology, Hospital Universitario Central de Asturias, Oviedo, Spain; 5 Division of Neurology, Department of Medicine, St. Michael’s Hospital, Toronto, Canada; 6 Clinical Outcomes and Decision Neuroscience Unit, Li Ka Shing Institute, University of Toronto, Toronto, Canada; Ladoke Akintola University of Technology Teaching Hospital: LAUTECH Teaching Hospital, NIGERIA

## Abstract

**Background:**

Decision-making in complex medical conditions is a cognitively demanding task influenced by clinician-specific behavioral factors. Procrastination, the voluntary delay of intended actions despite foreseeable adverse outcomes, is a self-regulation failure often exacerbated by high-stress clinical environments. This study evaluated the prevalence of procrastination among healthcare professionals (HCPs) managing spinal muscular atrophy (SMA) and analyzed its associations with burnout and healthcare-related regret.

**Methods:**

We conducted a non-interventional, cross-sectional, web-based study of HCPs recruited through the Spanish CuidAME registry. Participants were assessed using a battery of validated instruments: the Pure Procrastination Scale (PPS), the Regret Intensity Scale (RIS-10), the Evidence-Based Practice Attitude Scale (EBPAS), and a single-item burnout measure. In addition to multivariate logistic regression, we employed a regression-based mediation analysis with bootstrap resampling to explore potential indirect effects of burnout on procrastination via regret intensity.

**Results:**

Thirty-seven HCPs completed the study. Moderate-to-high procrastination was identified in 35.1% of the cohort. PPS scores correlated significantly with burnout (rho = 0.49, p = 0.002) and regret intensity (rho = 0.43, p = 0.007). Multivariate analysis identified burnout as the single independent factor associated with procrastination (OR: 8.17; 95%CI: 1.60–41.62; p = 0.011). Although burnout significantly predicted increased regret intensity, mediation analysis confirmed no significant indirect effect (beta = 0.98; 95%CI: −1.74 to 3.79). Burnout maintained a robust association with procrastination, independent of the mediator (beta = 5.11, p = 0.039).

**Conclusions:**

Procrastination is a prevalent behavioral trait in SMA care. While procrastination correlates with healthcare-related regret, burnout serves as its primary independent predictor of this behavior. These exploratory findings suggest that targeted interventions to mitigate clinician burnout may facilitate the optimization of decision-making processes in complex neuromuscular care.

## Introduction

Spinal Muscular Atrophy (SMA) is a progressive, autosomal recessive neuromuscular disorder caused by mutations in the SMN1 gene, resulting in motor neuron degeneration and profound muscle atrophy [1,2]. The disease manifests as a complex multisystem condition involving motor, respiratory, orthopedic, and nutritional complications that significantly impact the quality of life for both patients and caregivers [1–3]. While the last decade has seen the advent of transformative disease-modifying therapies that have fundamentally altered the prognosis of SMA, shifting it from a fatal, progressive neuromuscular disorder to a treatable condition with improved survival and motor function, healthcare professionals (HCPs) managing the disease continue to operate within a landscape of significant stress and clinical uncertainty [1–5]. This environment is shaped by evolving clinical phenotypes and persistent real-world challenges, including strength-gain plateaus, chronic fatigability, and critical bulbar impairment [3,4].

Decision-making in complex neurological conditions is a cognitively and emotionally demanding process [6]. Beyond institutional and regulatory frameworks, clinician-specific cognitive and behavioral characteristics are increasingly recognized as pivotal determinants of innovation adoption and adherence to clinical guidelines [7–10]. Regret, a cognitive emotion rooted in counterfactual thinking and the conviction that an alternative choice would have yielded superior outcomes, serves as a primary drive of human behavior [11]. In medical settings, the emotional sequelae of past clinical decisions profoundly influence subsequent choices [11]. Clinicians experiencing intense regret often adopt avoidance behaviors as a psychological defense mechanism. To mitigate this emotional burden, HCPs may exhibit behavioral biases, most notably procrastination [12–15]. Defined as the intentional and unnecessary delay of intended action despite awareness of potential negative consequences, procrastination represents a failure of self-regulation rather than a deficit in time management [7].

Recent data indicate that therapeutic inertia, the failure to initiate or intensify therapy when clinically indicated, affects more than one-third of treatment decisions in SMA care [16]. This inertia appears to be driven primarily by clinician-specific behavioral preferences rather than a lack of scientific evidence or knowledge gaps [16]. While clinician burnout is a well-documented phenomenon in neurology, its specific role as a correlate of procrastination and suboptimal decision-making in SMA remains inadequately researched [17,18].

Addressing the knowledge-to-action gap in neuromuscular medicine requires a granular understanding of the relationships between burnout, healthcare-related regret, and procrastination. Identifying these behavioral barriers is vital to optimizing outcomes in a field where early intervention is the primary determinant of survival and motor function preservation [1,2,5]. The primary objective of this study was to assess the prevalence of procrastination within a multidisciplinary cohort of HCPs managing SMA in Spain. Furthermore, we aimed to elucidate the mechanistic interplay between this behavioral bias and key psychological determinants, specifically burnout, regret, and attitudes toward evidence-based innovation.

## Methods

We conducted a non-interventional, cross-sectional, we-base study to evaluate the behavioral and psychological profiles of HCPs managing SMA. Participants with clinical expertise in SMA were recruited via the CuidAME registry, a national platform dedicated to SMA research and specialized care [19]. This network registry comprises 148 HCPs practicing across 42 hospitals throughout Spain, providing a representative cross-section of the country’s SMA specialist landscape.

The study was conducted according to the guidelines of the Declaration of Helsinki, and approved by the Institutional Review Board of Hospital Universitario Clínico San Carlos in Madrid, Spain. A written informed consent was obtained from all subjects involved in the study.

### Data collection and study measurements

Participants data were acquired via a structured electronic survey designed to capture demographics, professional background, and practice environment characteristics (December 2024 to June 2025). To characterize the participants’ behavioral factors, we utilized a battery of validated psychometric instruments. General procrastination tendencies were assessed using the Pure Procrastination Scale (PPS), a 12-item tool with total scores ranging from 12 to 60 [20]. Moderate-to-high procrastination was defined as a total score between 33 and 60 [21]. This threshold is consistent with prior research identifying significant procrastination tendencies in adult populations. The Regret Intensity Scale (RIS-10) was employed to evaluate emotional sequelae from past clinical events [22]. Participants rated their current feelings across 10 items on a 5-point Likert scale (1, strongly disagree; 5, strongly agree), with scores exceeding 30 classified as moderate-to-high regret. Burnout was measured using a validated single-item instrument from the Physician Work Life Study [23]. This 5-point ordinal scale ranges from 1 (“I enjoy my work; I have no symptoms of burnout”) to 5 (“I feel completely burned out and often wonder if I can go on”). Scores of 3 or higher were indicative of burnout. Attitudes toward evidence-based innovations were assessed via the Evidence-Based Practice Attitude Scale (EBPAS) [24]. This 15-item tool (score range: 0–60) utilizes a 5-point Likert scale (0, not at all; 4, to a very great extent). A high willingness to adopt innovations was defined as a score at or above the 75^th^ percentile of the cohort distribution. Patient-oriented empathy was measured with the Jefferson Scale of Empathy-Health Professionals (JSE), comprising 20 items on a 7-point Likert scale [25]. Total scores range from 20 to 140, with higher values reflecting greater empathic engagement. Professional risk-taking attitudes were evaluated using a validated question of the German Socio-Economic Panel [26]. Participants rated their willingness to take risks at work on scale from 0 (“not at all willing”) to 10 (“very willing”), with the 75^th^ percentile serving as the threshold for a high-risk attitude.

### Statistical analysis

Descriptive statistics were employed to summarize the cohort’s characteristics. Categorical variables are expressed as frequencies and percentages, while continuous variables are presented as means with standard deviations (SD) or medians with interquartile ranges (IQR), as appropriate. Intergroup comparisons utilized Chi-square or Fisher’s exact tests for categorical data and the Mann-Whitney U test for continuous variables.

The association between moderate-to-high procrastination, the primary dependent variable, and potential independent predictors was evaluated using binary logistic regression. To identify independent predictors, variables demonstrating a p-value <0.20 in bivariate analysis were entered into a final multivariate model using backward selection. Additionally, we assessed correlations between procrastination (PPS score), healthcare-related regret (RIS-10), burnout, and openness to innovation (EBPAS) using Spearman´s rank correlation coefficient (rho).

**Fig 1:**
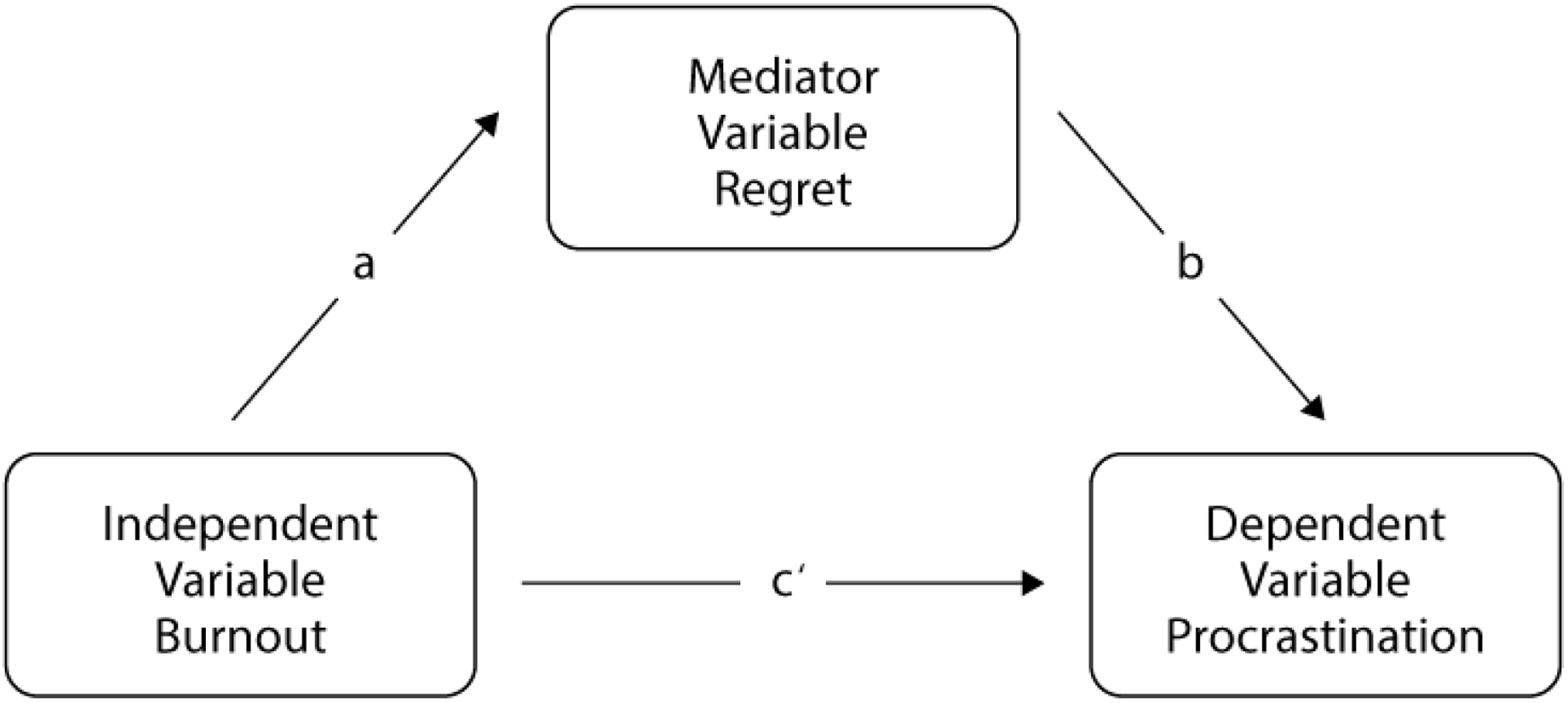
Model plot of mediation between burnout, procrastination, and regret.

To investigate the mechanistic relationship between clinician-specific factors, we performed a regression-based mediation analysis within the potential outcomes framework. Utilizing the PROCESS macro for SPSS (Model 4), we evaluated the indirect effect of burnout (independent variable, X) on procrastination (dependent variable, Y) through regret intensity (mediator, M). This analysis estimated three primary ordinary least squares (OLS) regression equations to map the following pathways: 1) path *a*: the effect of burnout on regret intensity, path *b*: the effect of regret intensity on procrastination, holding burnout constant, and path *c’* (direct effect): the residual impact of burnout on procrastination after accounting for the mediator (Fig 1). The total effect (path *c*) represented the impact of burnout on procrastination without the mediator in the model.

Given the modest sample size, we employed non-parametric bootstrapping with 1,000 resamples to generate 95% bias-corrected confidence intervals (CIs) for the indirect effect (*a* x *b*). Statistical significance for the indirect effect was determined if the 95% CI excluded zero.

Considering the exploratory nature of this study and the restricted sample size, no formal adjustments for multiple testing were applied. This approach was adopted to minimize the risk of Type II errors and to ensure the identification of potentially relevant behavioral signals within this highly specialized cohort. Consequently, results are interpreted based on their clinical significance and the consistency of associations across diverse psychometric measures.

## Results

A total of 37 HCPs completed the study (62.2% female), representing a 25% response rate from the national registry. The multidisciplinary cohort included pediatric neurologists (37.8%), neurologists (21.6%), speech-language therapists (16.2%), and rehabilitation specialists (8.1%). The majority (70.3%) practiced in academic hospitals, and 86.5% worked with a multidisciplinary team. Participants had a median of 8.0 years (IQR: 5.0–13.5) of clinical experience in SMA care. Further details are summarized in Table 1.

**Table 1:** Main characteristics of the participants.

	Total N = 37	Moderate-highprocrastination N = 13	Rest of the sample N = 24	p-value
Sex, female, n (%)	23 (62.2)	7 (55.8)	16 (66.7)	0.495
Experience managing SMA, years, median (IQR)	8.0 (5.0-13.5)	10.0 (5.0-12.0)	7.0 (5.0-16.5)	0.761
Investigator in clinical trials, n (%)	23 (62.2)	10 (76.9)	13 (54.2)	0.238
Author of peer-reviewed publications, n (%)	19 (51.4)	7 (53.8)	12 (50.0)	0.823
Type of hospital, academic, n (%)	26 (70.3)	8 (61.5)	18 (75.0)	0.637
Work in a multidisciplinary team, n (%)	32 (86.5)	11 (84.6)	21 (87.5)	0.923
PPS score, mean (SD)	29.6 (8.3)	38.5 (4.7)	24.8 (5.4)	<0.001
RIS-10 score, mean (SD)	18.6 (7.9)	23.2 (8.5)	16.2 (6.4)	0.009
Moderate-to-high regret intensity, n (%)	4 (10.8)	3 (23.1)	1 (4.2)	0.115
EBPAS score, mean (SD)	35.2 (6.4)	34.1 (6.0)	35.8 (6.8)	0.483
High openness to innovation, n (%)	10 (27.0)	1 (7.7)	9 (37.5)	0.065
Risk attitude, n (%)	9 (24.3)	1 (7.7)	8 (33.3)	0.119
JSE score, mean (SD)	124.8 (10.6)	125.6 (9.3)	124.4 (11.3)	0.738
High empathy, n (%)	17 (45.9)	6 (46.2)	11 (45.8)	0.985
Burnout, n (%)	10 (27.0)	7 (53.8)	3 (12.5)	0.017

EBPAS = Evidence-Based Practice Attitude Scale; IQR = Interquartile range; JSE = Jefferson Scale of Empathy; PPS = Pure Procrastination Scale; RIS-10 = Regret Intensity Scale; SD = Standard deviation.

Moderate-to-high procrastination was identified in 35.1% of participants, with a mean PPS score of 29.6 (SD: 8.3). The prevalence of the other relevant behavioral traits observed in the cohort included high empathy (45.9%), burnout (27.0%), and moderate-to-high regret intensity (10.8%) (Table 1).

HCPs in the moderate-to-high procrastination group reported significantly greater regret intensity (mean RIS-10 score: 23.2 [8.5] vs. 16.2 [6.4]; p = 0.009) and a higher prevalence of burnout (p = 0.017) compared to the low procrastination group (Table 1). HCPs characterized by high openness to innovation (27%), reported significantly lower mean procrastination scores compared to those with lower openness (mean PPS score: 24.7 [7.6] vs. 31.4 [8.0]; p = 0.030).

In the multivariate logistic regression analysis, the presence of burnout was associated with moderate-to-high procrastination (OR: 8.17; 95% CI: 1.60–41.62; p = 0.011). Procrastination scores demonstrated significant positive correlations with both burnout (rho = 0.499, p = 0.002) and regret intensity (rho = 0.439, p = 0.007) (Table 2).

**Table 2:** Spearman correlations between Pure Procrastination Scale and study outcome measures.

	rho (p-value)
Burnout	0.49 (0.002)
RIS-10 score	0.43 (0.007)
EBPAS score	−0.25 (0.126)
Risk attitude	−0.05 (0.741)
JSE score	−0.03 (0.837)

EBPAS = Evidence-Based Practice Attitude Scale; JSE = Jefferson Scale of Empathy; RIS-10 = Regret Intensity Scale; rho = Spearman’s rank correlation coefficient.

We performed a regression-based mediation analysis to investigate the mechanistic relationship between burnout (X), healthcare regret (M), and procrastination (Y) (Table 3). Path *a*: Burnout was significantly associated with increased healthcare regret intensity (beta = 7.36, SE = 1.59; p < 0.001). Path *b*: Regret intensity did not significantly predict procrastination when controlling for burnout (beta = 0.13, SE = 0.19; p = 0.506). Total effect (path *c*): In the absence of the mediator, burnout was a significant predictor of procrastination (beta = 6.10, SE = 1.86; p = 0.002). Direct effect (path *c’*): After accounting for regret intensity, the direct impact of burnout on procrastination remained statistically significant (beta = 5.11, SE = 2.38; p = 0.039). To further explore this mechanism, the indirect effect of burnout on procrastination via regret intensity was evaluated using a bootstrapping procedure. While the point estimate was 0.9872, the bias-corrected bootstrap 95% CI contained zero (−1.74 to 3.79), indicating that the indirect effect was not statistically significant.

**Table 3:** Mediation analysis between burnout, procrastination, and healthcare regret intensity.

Analysis Path	Coefficient (β)	Standard Error	p-value	95% CI
Path *a* (Burnout – Regret)	7.36	1.59	<0.001	4.13, 10.59
Path *b* (Regret – Procrastination)	0.13	0.19	0.506	−0.27, 0.53
Total Effect (Path *c*)	6.10	1.86	0.002	2.31, 9.89
Direct Effect (Path *c’*)	5.11	2.38	0.039	0.25, 9.96
Indirect Effect (a x b)	0.98	1.40 (BootSE)	–	−1.74, 3.79*

*Statistically non-significant as the 95% bias-corrected bootstrap confidence interval contains zero.

## Discussion

The individualized management of patients with SMA has entered an increasingly complex era following the approval of disease-modifying therapies [1,2]. While early intervention remains the primary determinant of survival and motor function preservation, clinicians must now navigate a shifting therapeutic ecosystem that extends far beyond initial treatment [1,2,5]. Physicians are responsible for managing persistent motor and systemic dysfunctions, often in the absence of standardized protocols for treatment switching and combination strategies [3–5].

Our study identified a 35.1% prevalence of moderate-to-high procrastination among HCPs managing SMA. Procrastination is defined as the voluntary and unnecessary delay of intended, critical tasks despite the anticipation of adverse consequences [12,27–29]. Historically, psychological research has conceptualized this behavior through the lens of delay discounting, wherein the immediate reward of an alternative activity is prioritized over the larger, albeit delayed, reward of task completion [15]. Within this behavioral framework, procrastination is often sustained via negative reinforcement. The abrupt attenuation of anxiety experienced when a task is finalized immediately prior to a deadline reinforces the cycle of delay [12,15].

While general prevalence estimates indicate that 15% to 25% of adults are chronic procrastinators, this behavior is increasingly recognized as a specific failure of self-regulation precipitated by high-stress environments [12,13,30]. According to the stress context vulnerability model, high-stress environments exhaust cognitive and emotional resources, thereby lowering the threshold for tolerating negative affective states [12]. This maladaptive coping mechanism is associated with diminished well-being, manifesting as elevated perceived stress, impaired sleep quality, and an increased incidence of somatic illness [12,29–32]. In the specialized context of SMA management, where diagnostic, therapeutic, and longitudinal monitoring protocols are exceptionally rigorous, procrastination may function as a low-resource emotion regulation strategy. In this setting, it serves a defensive response to mitigate the immediate negative effect associated with the high cognitive load and demanding nature of clinical duties [12,29].

A primary finding of this study is the robust predictive power of burnout, with HCPs experiencing exhaustion being 8.17 times more likely to report moderate-to-high procrastination. This finding aligns with conservation of resources theory, which posits that clinicians voluntarily reduce effort as a defensive response to prevent further energy depletion [12,33]. In our cohort, burnout demonstrated a robust independent association with behavioral delay. This mirrors a broader crisis in neurology, where burnout prevalence frequently exceed 60% [17,18].

From a theoretical perspective, this association may be interpreted through the ‘amygdala hijack’ framework [12,15]. In this hypothesis-generating model, professional exhaustion depletes the prefrontal cortex, which then fails to modulate the limbic drive for immediate emotional relief [12,15,34]. Recent neuro-computational models further suggest that this behavior stems from a cognitive bias characterized by the temporal discounting of effort; specifically, the anticipated cost of a task is attenuated by delay while the perceived reward remains stable [35].

In the management of SMA, the perceived “vividness” of immediate effort, including coordinating multidisciplinary care and addressing the profound concerns of patients and caregivers, can trigger a preference for decision-making delay. The immediate aversiveness of these high-stakes clinical responsibilities may outweigh the anticipated rewards of long-term survival and motor function preservation, which appear cognitively more temporal and distant. Consequently, clinicians may enter a cycle of delay maintained by negative reinforcement, where the transient relief from aversive tasks reinforces future therapeutic inertia. While our findings confirm a correlation between burnout and behavioral delay, further neurofunctional research is required to definitively link these clinical observations to specific limbic-prefrontal mechanisms.

Although we hypothesized that healthcare regret would mediate the relationship between burnout and procrastination, our mediation analysis revealed a more nuanced relationship. Our findings demonstrate that burnout exerts a powerful direct effect on procrastination that is not explained by the intensity of regret. While the mediation was not statistically significant, the correlation between procrastination and regret remains clinically relevant (rho = 0.43, p = 0.007). The highly significant path *a* in our model (beta = 7.36, p < 0.001) indicates that burnout significantly intensifies the experience of regret. This suggests that regret and procrastination may represent parallel manifestations of a resource-depleted environment rather than a linear causal chain.

This mediation analysis should be considered exploratory. Although the model provided a framework for understanding potential mechanistic pathways, the analysis was likely underpowered to detect subtle indirect effects, as evidenced by non-significant bootstrap confidence intervals. These findings are intended to be hypothesis-generating, suggesting that the psychological burden in SMA care may involve parallel stress manifestations.

Furthermore, higher procrastination scores were significantly associated with reduced openness to innovations, suggesting that decision-making delay acts as a systemic barrier to evidence-based practice. This is consistent with temporal motivation theory, which suggests that the perceived aversiveness of complex new protocols facilitates delay when clinical rewards appear distant. Interestingly, while 86.5% of our cohort practiced within multidisciplinary teams, this structure was not associated with lower procrastination levels in our sample. However, as only 13.5% of participants practiced outside such teams, further research in diverse settings is required to determine if collaborative environments can effectively buffer individual self-regulation failures.

Addressing these barriers requires a two-tiered approach. At the organizational level, interventions should aim to reduce the burden associated with care delivery, administrative load and ‘red tape’, particularly critical in the complex management of neuromuscular diseases, where shortages of specialized healthcare professionals further intensify workload and complicate clinical decision-making [36]. Such measures may include the integration of support services (e.g., social work and counselling) to alleviate non-clinical responsibilities [37]. At individual-level, targeted training in mindfulness and emotion-regulation may help restore self-regulatory capacity and enhance clinicians’ resilience [18,38].

This study has several limitations that warrant consideration. First, the reliance on self-reported measures introduces potential recall and social desirability biases. In high-responsibility professions, such biases may lead to an underestimation of procrastination prevalence. Furthermore, to minimize respondent burden and optimize participation, we utilized a validated single-item burnout measure rather than the multidimensional Maslach Burnout Inventory. Consequently, our findings provide a global assessment of burnout rather than a granular characterization of its distinct psychological domains. Second, although the sample size appears modest, it was recruited via the national CuidAME registry. This cohort represents 25% of the 148 registered SMA specialists in Spain, providing a representative cross-section of the multidisciplinary workforce in this specialized field. Despite the voluntary nature of the survey, the high proportion of participants practicing in academic hospitals (70.3%) and multidisciplinary teams (86.5%) accurately reflects the current Spanish standard of care for SMA. Nevertheless, the small absolute sample size likely limited the statistical power to identify subtle associations and contributed to the wide confidence intervals observed in our multivariate analysis. To mitigate the risk of overfitting and model instability inherent in small-cohort studies, we employed a conservative backward selection process. Third, several variables and mechanistic pathways warrant further investigation in more expansive cohorts. Although specialized clinical experience served as a viable proxy for professional background, the absence of chronological age in our data collection remains an acknowledged limitation. Furthermore, the current sample size precluded a granular sub-analysis of clinical demands, such as patient volume or SMA type distribution, as stratifying the cohort by these variables would have yielded statistically underpowered comparisons. Finally, the mediation analysis exploring the interplay between burnout, regret intensity, and procrastination must also be considered exploratory; as evidenced by the non-significant bootstrap confidence intervals, the study was likely underpowered to detect subtle indirect effects. Crucially, the cross-sectional design of this study precludes the establishment of definitive causal links. It is plausible that the relationship between these factors is bidirectional: while professional exhaustion may facilitate behavioral delay, chronic procrastination may simultaneously exacerbate stress and anxiety, ultimately contributing to the development of burnout. These findings are therefore intended to be hypothesis-generating, suggesting that the psychological burden in SMA care involves complex, parallel manifestations of stress rather than a simple linear causal chain. Despite these constraints, the robust predictive power of burnout identified in our multivariate model (OR: 8.17) indicates that professional exhaustion is a primary independent correlate of behavioral delay. Future research utilizing larger, multi-national registries is warranted to elucidate the complex interactions between patient-specific demands and these internal behavioral barriers.

## Conclusion

This exploratory study indicates that procrastination is a prevalent behavioral trait among HCPs managing SMA in Spain. While procrastination, burnout, and healthcare-related regret appear highly interrelated, our multivariate analysis identifies burnout as a significant independent correlate of behavioral delay. These findings suggest that clinician-specific psychological factors may function as internal barriers to the adoption of evidence-based, personalized medicine. Given the study’s methodological constraints, these results should be interpreted as hypothesis-generating. Nevertheless, they underscore the clinical importance of mitigating clinician burnout as a potential strategy to optimize decision-making processes in complex neuromuscular care.
